# PP17 Evidence And Economic Models Used By CONITEC In The Evaluation And Incorporation Of Medical Devices In The Unified Health System

**DOI:** 10.1017/S0266462325102377

**Published:** 2025-12-29

**Authors:** Aldenora Maria Ximenes Rodrigues, Mônica Vinhas de Souza, Katia Elizabete Galdino, Ketinlly Yasmyne Nascimento Martins

## Abstract

**Introduction:**

Health technology assessment (HTA), conducted by the National Committee for Health Technology Incorporation (CONITEC), guides the incorporation of technologies into the Unified Health System (SUS). This process is challenging because it requires diverse evidence and applying appropriate economic methods to the national context. This study analyzed the types of evidence and economic models used in medical device assessment reports, identifying gaps and opportunities for improvement.

**Methods:**

Twenty-one reports published on the CONITEC Recommendations Panel between 2012 and 2021 were analyzed using the filters “Technology Type – Product” and “Ministry of Health Decision – Incorporate.” We reviewed the reports to identify: (i) types of evidence used, including public consultations, patient perspectives, and technology horizon monitoring (THM); (ii) applied economic models; and (iii) described limitations. The extracted data were categorized and analyzed to assess patterns, frequencies, and methodological gaps.

**Results:**

Of the reports analyzed, 85.7 percent included systematic reviews and meta-analyses, and 52.4 percent cited observational studies or randomized clinical trials. Most reports conducted public consultations, while fewer than 20 percent considered patient perspectives. THM appeared in 14.3 percent of the reports. There was no use of real-world evidence or evaluation of wearable devices. In economic models, 71.4 percent applied cost-effectiveness analysis, while 42.9 percent of reports lacked detailed economic analysis. Common gaps included reliance on international data and a need for more local information, impacting the accuracy of evaluations.

**Conclusions:**

Brazil can improve HTA by including more real-world evidence, using THM more substantially, and considering patient perspectives more broadly. Enhancing the systematization of evidence searches and prioritizing local data would reduce uncertainties, aligning CONITEC’s practices with global next-generation evidence trends, leading to more effective and contextually relevant decisions.

